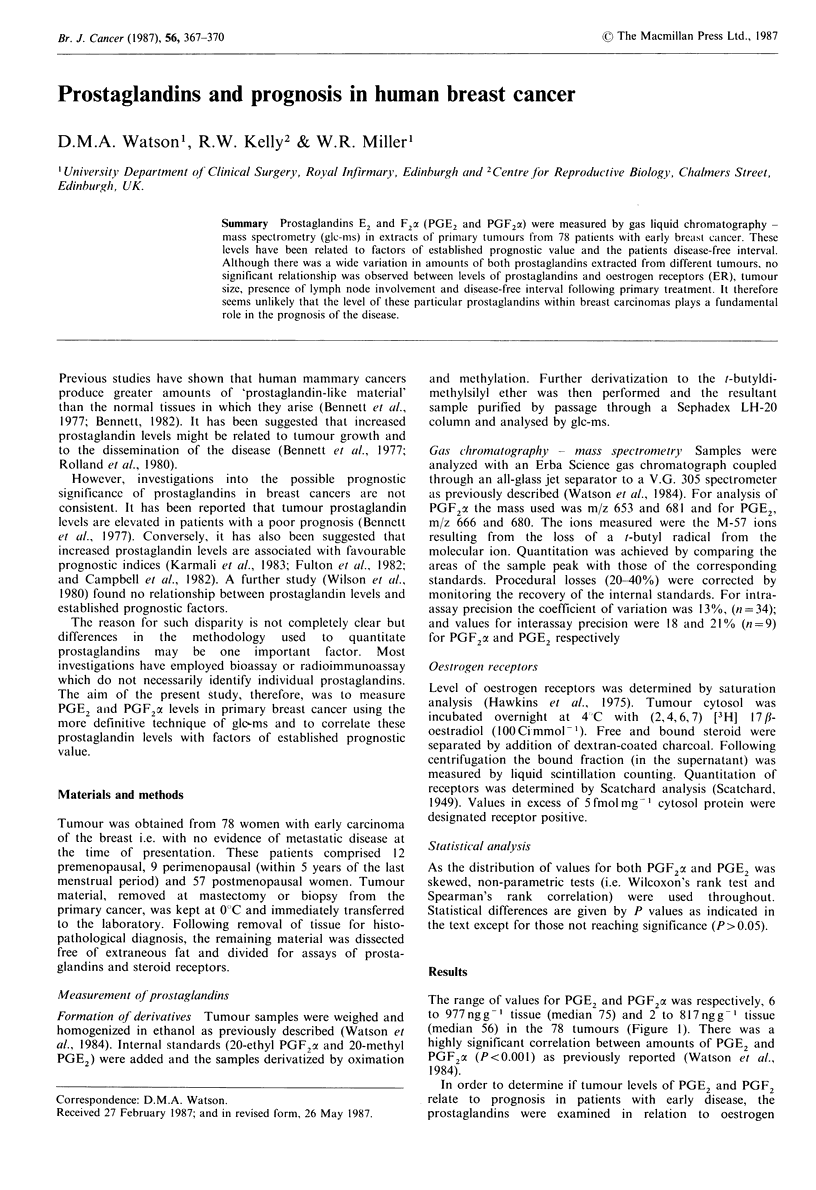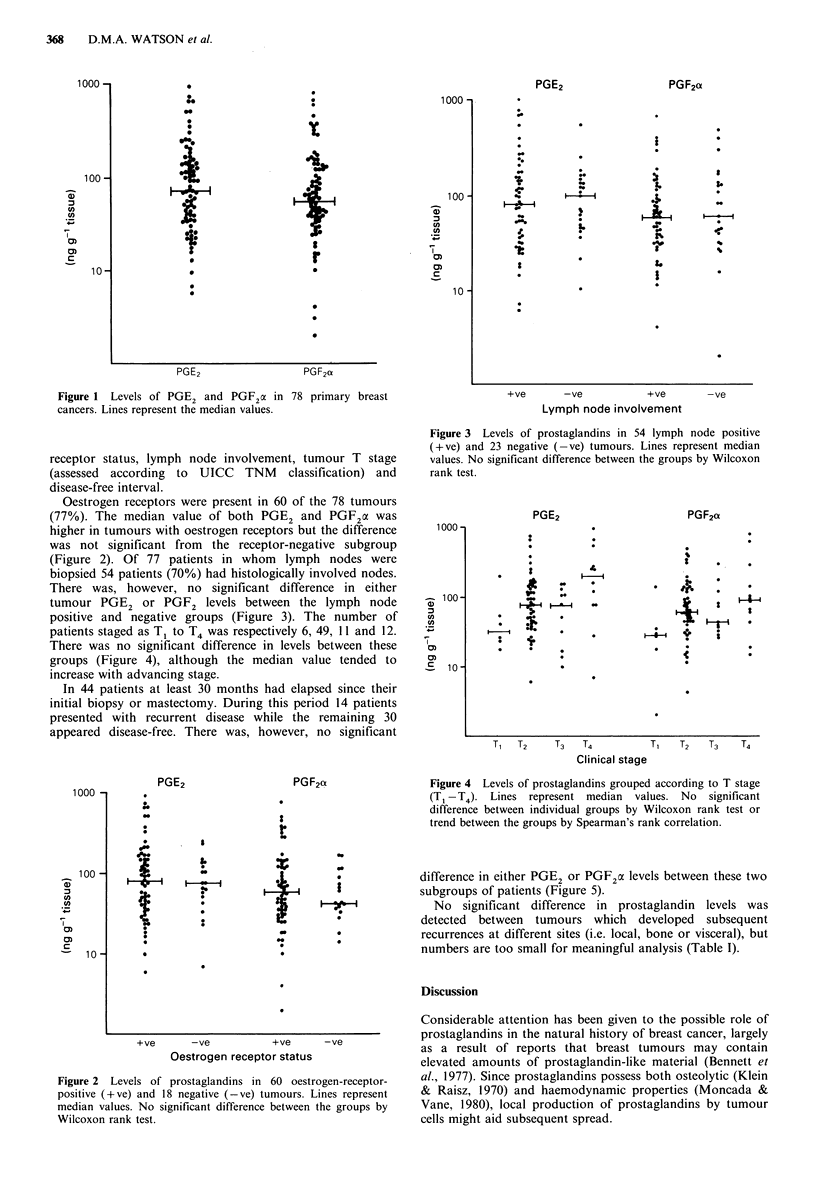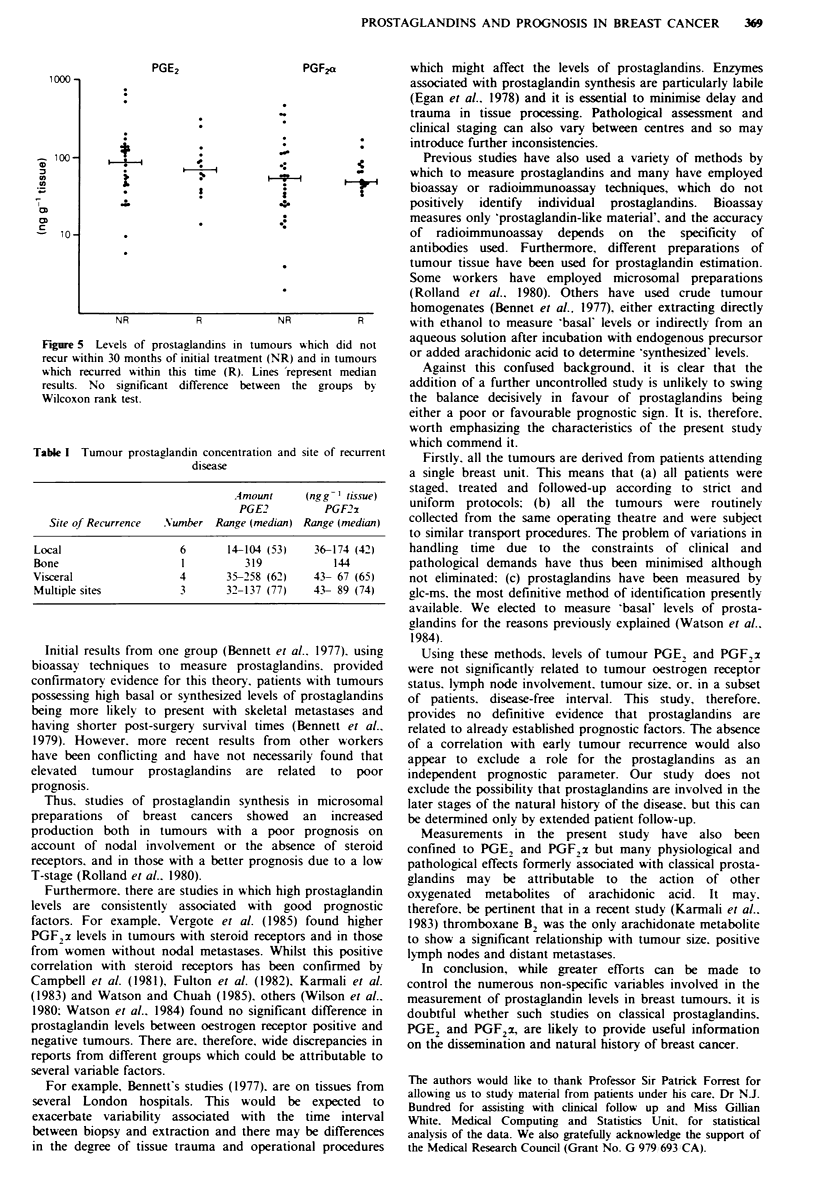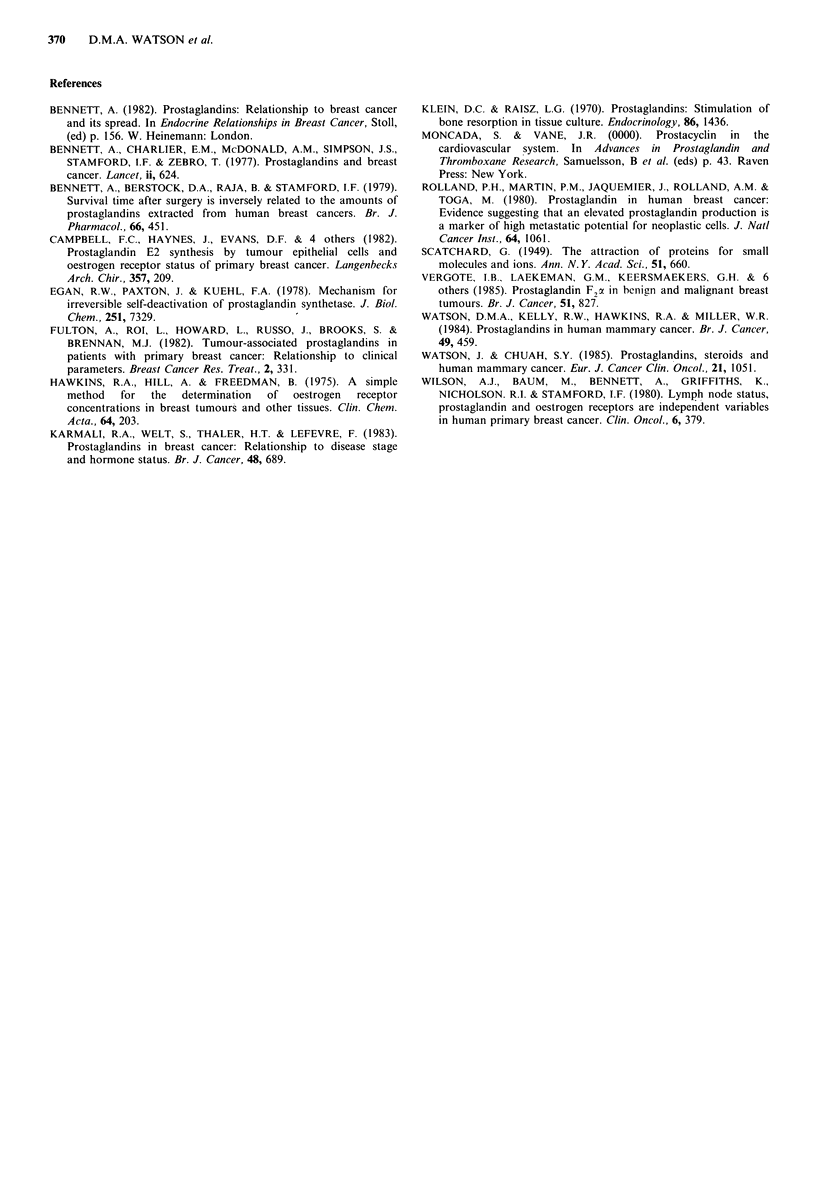# Prostaglandins and prognosis in human breast cancer.

**DOI:** 10.1038/bjc.1987.204

**Published:** 1987-09

**Authors:** D. M. Watson, R. W. Kelly, W. R. Miller

**Affiliations:** University Department of Clinical Surgery, Royal Infirmary, Edinburgh, UK.

## Abstract

Prostaglandins E2 and F2 alpha (PGE2 and PGF2 alpha) were measured by gas liquid chromatography--mass spectrometry (glc-ms) in extracts of primary tumours from 78 patients with early breast cancer. These levels have been related to factors of established prognostic value and the patients disease-free interval. Although there was a wide variation in amounts of both prostaglandins extracted from different tumours, no significant relationship was observed between levels of prostaglandins and oestrogen receptors (ER), tumour size, presence of lymph node involvement and disease-free interval following primary treatment. It therefore seems unlikely that the level of these particular prostaglandins within breast carcinomas plays a fundamental role in the prognosis of the disease.


*Br. J. Cancer* (1987), **56**, 367–370 © The Macmillan Press Ltd., 1987

### Prostaglandins and prognosis in human breast cancer

D.M.A. Watson[1], R.W. Kelly[2] & W.R. Miller[1]

[1]*University Department of Clinical Surgery, Royal Infirmary, Edinburgh and* [2]*Centre for Reproductive Biology, Chalmers Street, Edinburgh, UK.*

**Summary** Prostaglandins $E_2$ and $F_2\alpha$ ($PGE_2$ and $PGF_2\alpha$) were measured by gas liquid chromatography – mass spectrometry (glc-ms) in extracts of primary tumours from 78 patients with early breast cancer. These levels have been related to factors of established prognostic value and the patients disease-free interval. Although there was a wide variation in amounts of both prostaglandins extracted from different tumours, no significant relationship was observed between levels of prostaglandins and oestrogen receptors (ER), tumour size, presence of lymph node involvement and disease-free interval following primary treatment. It therefore seems unlikely that the level of these particular prostaglandins within breast carcinomas plays a fundamental role in the prognosis of the disease.

Previous studies have shown that human mammary cancers produce greater amounts of 'prostaglandin-like material' than the normal tissues in which they arise (Bennett *et al.*, 1977; Bennett, 1982). It has been suggested that increased prostaglandin levels might be related to tumour growth and to the dissemination of the disease (Bennett *et al.*, 1977; Rolland *et al.*, 1980).

However, investigations into the possible prognostic significance of prostaglandins in breast cancers are not consistent. It has been reported that tumour prostaglandin levels are elevated in patients with a poor prognosis (Bennett *et al.*, 1977). Conversely, it has also been suggested that increased prostaglandin levels are associated with favourable prognostic indices (Karmali *et al.*, 1983; Fulton *et al.*, 1982; and Campbell *et al.*, 1982). A further study (Wilson *et al.*, 1980) found no relationship between prostaglandin levels and established prognostic factors.

The reason for such disparity is not completely clear but differences in the methodology used to quantitate prostaglandins may be one important factor. Most investigations have employed bioassay or radioimmunoassay which do not necessarily identify individual prostaglandins. The aim of the present study, therefore, was to measure $PGE_2$ and $PGF_2\alpha$ levels in primary breast cancer using the more definitive technique of glc-ms and to correlate these prostaglandin levels with factors of established prognostic value.

### Materials and methods

Tumour was obtained from 78 women with early carcinoma of the breast i.e. with no evidence of metastatic disease at the time of presentation. These patients comprised 12 premenopausal, 9 perimenopausal (within 5 years of the last menstrual period) and 57 postmenopausal women. Tumour material, removed at mastectomy or biopsy from the primary cancer, was kept at 0°C and immediately transferred to the laboratory. Following removal of tissue for histopathological diagnosis, the remaining material was dissected free of extraneous fat and divided for assays of prostaglandins and steroid receptors.

### *Measurement of prostaglandins*

*Formation of derivatives* Tumour samples were weighed and homogenized in ethanol as previously described (Watson *et al.*, 1984). Internal standards (20-ethyl $PGF_2\alpha$ and 20-methyl $PGE_2$) were added and the samples derivatized by oximation and methylation. Further derivatization to the *t*-butyldimethylsilyl ether was then performed and the resultant sample purified by passage through a Sephadex LH-20 column and analysed by glc-ms.

*Gas chromatography – mass spectrometry* Samples were analyzed with an Erba Science gas chromatograph coupled through an all-glass jet separator to a V.G. 305 spectrometer as previously described (Watson *et al.*, 1984). For analysis of $PGF_2\alpha$ the mass used was m/z 653 and 681 and for $PGE_2$, m/z 666 and 680. The ions measured were the M-57 ions resulting from the loss of a *t*-butyl radical from the molecular ion. Quantitation was achieved by comparing the areas of the sample peak with those of the corresponding standards. Procedural losses (20–40%) were corrected by monitoring the recovery of the internal standards. For intra-assay precision the coefficient of variation was 13%, ($n=34$); and values for interassay precision were 18 and 21% ($n=9$) for $PGF_2\alpha$ and $PGE_2$ respectively

### *Oestrogen receptors*

Level of oestrogen receptors was determined by saturation analysis (Hawkins *et al.*, 1975). Tumour cytosol was incubated overnight at 4°C with (2,4,6,7) [$^3$H] 17$\beta$-oestradiol (100 Ci mmol$^{-1}$). Free and bound steroid were separated by addition of dextran-coated charcoal. Following centrifugation the bound fraction (in the supernatant) was measured by liquid scintillation counting. Quantitation of receptors was determined by Scatchard analysis (Scatchard, 1949). Values in excess of 5 fmol mg$^{-1}$ cytosol protein were designated receptor positive.

### *Statistical analysis*

As the distribution of values for both $PGF_2\alpha$ and $PGE_2$ was skewed, non-parametric tests (i.e. Wilcoxon's rank test and Spearman's rank correlation) were used throughout. Statistical differences are given by *P* values as indicated in the text except for those not reaching significance ($P>0.05$).

### Results

The range of values for $PGE_2$ and $PGF_2\alpha$ was respectively, 6 to 977 ng g$^{-1}$ tissue (median 75) and 2 to 817 ng g$^{-1}$ tissue (median 56) in the 78 tumours (Figure 1). There was a highly significant correlation between amounts of $PGE_2$ and $PGF_2\alpha$ ($P<0.001$) as previously reported (Watson *et al.*, 1984).

In order to determine if tumour levels of $PGE_2$ and $PGF_2$ relate to prognosis in patients with early disease, the prostaglandins were examined in relation to oestrogen

Correspondence: D.M.A. Watson.
Received 27 February 1987; and in revised form, 26 May 1987.

**Figure 1** Levels of $PGE_2$ and $PGF_2\alpha$ in 78 primary breast cancers. Lines represent the median values.

receptor status, lymph node involvement, tumour T stage (assessed according to UICC TNM classification) and disease-free interval.

Oestrogen receptors were present in 60 of the 78 tumours (77%). The median value of both $PGE_2$ and $PGF_2\alpha$ was higher in tumours with oestrogen receptors but the difference was not significant from the receptor-negative subgroup (Figure 2). Of 77 patients in whom lymph nodes were biopsied 54 patients (70%) had histologically involved nodes. There was, however, no significant difference in either tumour $PGE_2$ or $PGF_2$ levels between the lymph node positive and negative groups (Figure 3). The number of patients staged as $T_1$ to $T_4$ was respectively 6, 49, 11 and 12. There was no significant difference in levels between these groups (Figure 4), although the median value tended to increase with advancing stage.

In 44 patients at least 30 months had elapsed since their initial biopsy or mastectomy. During this period 14 patients presented with recurrent disease while the remaining 30 appeared disease-free. There was, however, no significant difference in either $PGE_2$ or $PGF_2\alpha$ levels between these two subgroups of patients (Figure 5).

**Figure 2** Levels of prostaglandins in 60 oestrogen-receptor-positive (+ve) and 18 negative (−ve) tumours. Lines represent median values. No significant difference between the groups by Wilcoxon rank test.

**Figure 3** Levels of prostaglandins in 54 lymph node positive (+ve) and 23 negative (−ve) tumours. Lines represent median values. No significant difference between the groups by Wilcoxon rank test.

**Figure 4** Levels of prostaglandins grouped according to T stage ($T_1-T_4$). Lines represent median values. No significant difference between individual groups by Wilcoxon rank test or trend between the groups by Spearman's rank correlation.

No significant difference in prostaglandin levels was detected between tumours which developed subsequent recurrences at different sites (i.e. local, bone or visceral), but numbers are too small for meaningful analysis (Table I).

### Discussion

Considerable attention has been given to the possible role of prostaglandins in the natural history of breast cancer, largely as a result of reports that breast tumours may contain elevated amounts of prostaglandin-like material (Bennett *et al.*, 1977). Since prostaglandins possess both osteolytic (Klein & Raisz, 1970) and haemodynamic properties (Moncada & Vane, 1980), local production of prostaglandins by tumour cells might aid subsequent spread.

**Figure 5** Levels of prostaglandins in tumours which did not recur within 30 months of initial treatment (NR) and in tumours which recurred within this time (R). Lines represent median results. No significant difference between the groups by Wilcoxon rank test.

**Table I** Tumour prostaglandin concentration and site of recurrent disease

| *Site of Recurrence* | *Number* | *Amount* *PGE2* *Range (median)* | *(ng g⁻¹ tissue)* *PGF2α* *Range (median)* |
|---|---|---|---|
| Local | 6 | 14–104 (53) | 36–174 (42) |
| Bone | 1 | 319 | 144 |
| Visceral | 4 | 35–258 (62) | 43– 67 (65) |
| Multiple sites | 3 | 32–137 (77) | 43– 89 (74) |

Initial results from one group (Bennett *et al.*, 1977), using bioassay techniques to measure prostaglandins, provided confirmatory evidence for this theory, patients with tumours possessing high basal or synthesized levels of prostaglandins being more likely to present with skeletal metastases and having shorter post-surgery survival times (Bennett *et al.*, 1979). However, more recent results from other workers have been conflicting and have not necessarily found that elevated tumour prostaglandins are related to poor prognosis.

Thus, studies of prostaglandin synthesis in microsomal preparations of breast cancers showed an increased production both in tumours with a poor prognosis on account of nodal involvement or the absence of steroid receptors, and in those with a better prognosis due to a low T-stage (Rolland *et al.*, 1980).

Furthermore, there are studies in which high prostaglandin levels are consistently associated with good prognostic factors. For example, Vergote *et al.* (1985) found higher $PGF_2\alpha$ levels in tumours with steroid receptors and in those from women without nodal metastases. Whilst this positive correlation with steroid receptors has been confirmed by Campbell *et al.* (1981), Fulton *et al.* (1982), Karmali *et al.* (1983) and Watson and Chuah (1985), others (Wilson *et al.*, 1980; Watson *et al.*, 1984) found no significant difference in prostaglandin levels between oestrogen receptor positive and negative tumours. There are, therefore, wide discrepancies in reports from different groups which could be attributable to several variable factors.

For example, Bennett's studies (1977), are on tissues from several London hospitals. This would be expected to exacerbate variability associated with the time interval between biopsy and extraction and there may be differences in the degree of tissue trauma and operational procedures which might affect the levels of prostaglandins. Enzymes associated with prostaglandin synthesis are particularly labile (Egan *et al.*, 1978) and it is essential to minimise delay and trauma in tissue processing. Pathological assessment and clinical staging can also vary between centres and so may introduce further inconsistencies.

Previous studies have also used a variety of methods by which to measure prostaglandins and many have employed bioassay or radioimmunoassay techniques, which do not positively identify individual prostaglandins. Bioassay measures only 'prostaglandin-like material', and the accuracy of radioimmunoassay depends on the specificity of antibodies used. Furthermore, different preparations of tumour tissue have been used for prostaglandin estimation. Some workers have employed microsomal preparations (Rolland *et al.*, 1980). Others have used crude tumour homogenates (Bennet *et al.*, 1977), either extracting directly with ethanol to measure 'basal' levels or indirectly from an aqueous solution after incubation with endogenous precursor or added arachidonic acid to determine 'synthesized' levels.

Against this confused background, it is clear that the addition of a further uncontrolled study is unlikely to swing the balance decisively in favour of prostaglandins being either a poor or favourable prognostic sign. It is, therefore, worth emphasizing the characteristics of the present study which commend it.

Firstly, all the tumours are derived from patients attending a single breast unit. This means that (a) all patients were staged, treated and followed-up according to strict and uniform protocols; (b) all the tumours were routinely collected from the same operating theatre and were subject to similar transport procedures. The problem of variations in handling time due to the constraints of clinical and pathological demands have thus been minimised although not eliminated; (c) prostaglandins have been measured by glc-ms, the most definitive method of identification presently available. We elected to measure 'basal' levels of prostaglandins for the reasons previously explained (Watson *et al.*, 1984).

Using these methods, levels of tumour $PGE_2$ and $PGF_2\alpha$ were not significantly related to tumour oestrogen receptor status, lymph node involvement, tumour size, or, in a subset of patients, disease-free interval. This study, therefore, provides no definitive evidence that prostaglandins are related to already established prognostic factors. The absence of a correlation with early tumour recurrence would also appear to exclude a role for the prostaglandins as an independent prognostic parameter. Our study does not exclude the possibility that prostaglandins are involved in the later stages of the natural history of the disease, but this can be determined only by extended patient follow-up.

Measurements in the present study have also been confined to $PGE_2$ and $PGF_2\alpha$ but many physiological and pathological effects formerly associated with classical prostaglandins may be attributable to the action of other oxygenated metabolites of arachidonic acid. It may, therefore, be pertinent that in a recent study (Karmali *et al.*, 1983) thromboxane $B_2$ was the only arachidonate metabolite to show a significant relationship with tumour size, positive lymph nodes and distant metastases.

In conclusion, while greater efforts can be made to control the numerous non-specific variables involved in the measurement of prostaglandin levels in breast tumours, it is doubtful whether such studies on classical prostaglandins, $PGE_2$ and $PGF_2\alpha$, are likely to provide useful information on the dissemination and natural history of breast cancer.

The authors would like to thank Professor Sir Patrick Forrest for allowing us to study material from patients under his care, Dr N.J. Bundred for assisting with clinical follow up and Miss Gillian White, Medical Computing and Statistics Unit, for statistical analysis of the data. We also gratefully acknowledge the support of the Medical Research Council (Grant No. G 979/693/CA).

### References

BENNETT, A. (1982). Prostaglandins: Relationship to breast cancer and its spread. In *Endocrine Relationships in Breast Cancer*, Stoll, (ed) p. 156. W. Heinemann: London.

BENNETT, A., CHARLIER, E.M., McDONALD, A.M., SIMPSON, J.S., STAMFORD, I.F. & ZEBRO, T. (1977). Prostaglandins and breast cancer. *Lancet*, **ii**, 624.

BENNETT, A., BERSTOCK, D.A., RAJA, B. & STAMFORD, I.F. (1979). Survival time after surgery is inversely related to the amounts of prostaglandins extracted from human breast cancers. *Br. J. Pharmacol.*, **66**, 451.

CAMPBELL, F.C., HAYNES, J., EVANS, D.F. & 4 others (1982). Prostaglandin E2 synthesis by tumour epithelial cells and oestrogen receptor status of primary breast cancer. *Langenbecks Arch. Chir.*, **357**, 209.

EGAN, R.W., PAXTON, J. & KUEHL, F.A. (1978). Mechanism for irreversible self-deactivation of prostaglandin synthetase. *J. Biol. Chem.*, **251**, 7329.

FULTON, A., ROI, L., HOWARD, L., RUSSO, J., BROOKS, S. & BRENNAN, M.J. (1982). Tumour-associated prostaglandins in patients with primary breast cancer: Relationship to clinical parameters. *Breast Cancer Res. Treat.*, **2**, 331.

HAWKINS, R.A., HILL, A. & FREEDMAN, B. (1975). A simple method for the determination of oestrogen receptor concentrations in breast tumours and other tissues. *Clin. Chem. Acta.*, **64**, 203.

KARMALI, R.A., WELT, S., THALER, H.T. & LEFEVRE, F. (1983). Prostaglandins in breast cancer: Relationship to disease stage and hormone status. *Br. J. Cancer*, **48**, 689.

KLEIN, D.C. & RAISZ, L.G. (1970). Prostaglandins: Stimulation of bone resorption in tissue culture. *Endocrinology*, **86**, 1436.

MONCADA, S. & VANE, J.R. (0000). Prostacyclin in the cardiovascular system. In *Advances in Prostaglandin and Thromboxane Research*, Samuelsson, B *et al.* (eds) p. 43. Raven Press: New York.

ROLLAND, P.H., MARTIN, P.M., JAQUEMIER, J., ROLLAND, A.M. & TOGA, M. (1980). Prostaglandin in human breast cancer: Evidence suggesting that an elevated prostaglandin production is a marker of high metastatic potential for neoplastic cells. *J. Natl Cancer Inst.*, **64**, 1061.

SCATCHARD, G. (1949). The attraction of proteins for small molecules and ions. *Ann. N.Y. Acad. Sci.*, **51**, 660.

VERGOTE, I.B., LAEKEMAN, G.M., KEERSMAEKERS, G.H. & 6 others (1985). Prostaglandin $F_2\alpha$ in benign and malignant breast tumours. *Br. J. Cancer*, **51**, 827.

WATSON, D.M.A., KELLY, R.W., HAWKINS, R.A. & MILLER, W.R. (1984). Prostaglandins in human mammary cancer. *Br. J. Cancer*, **49**, 459.

WATSON, J. & CHUAH, S.Y. (1985). Prostaglandins, steroids and human mammary cancer. *Eur. J. Cancer Clin. Oncol.*, **21**, 1051.

WILSON, A.J., BAUM, M., BENNETT, A., GRIFFITHS, K., NICHOLSON. R.I. & STAMFORD, I.F. (1980). Lymph node status, prostaglandin and oestrogen receptors are independent variables in human primary breast cancer. *Clin. Oncol.*, **6**, 379.